# Effect of Thermal Processes on *S*-Allyl Cysteine Content in Black Garlic

**DOI:** 10.3390/foods12061227

**Published:** 2023-03-13

**Authors:** Kanokwan Manoonphol, Uthaiwan Suttisansanee, Chadamas Promkum, Chaniphun Butryee

**Affiliations:** 1 Doctor of Philosophy Program in Nutrition, Faculty of Medicine Ramathibodi Hospital and Institute of Nutrition, Mahidol University, Bangkok 10400, Thailand; 2 Food and Nutrition Academic and Research Cluster, Institute of Nutrition, Mahidol University, Nakhon Pathom 73170, Thailand

**Keywords:** fermentation, antioxidant activity, phenolics, relative humidity, thermal treatment, Thai garlic, Chinese garlic, value-added

## Abstract

As a key component of black garlic (BG) products, S-allyl cysteine (SAC) is useful in reducing oxidative stress and inflammation. Several BG products with a high SAC content have been developed by thermal processing; however, the optimum conditions for thermal treatment for producing Thai garlic (multicloves) with a high SAC content compared to Chinese garlic (single clove) are still unknown. Moreover, the mechanism underlying the increase in SAC content in BG is unclear. Thus, this study aimed to investigate the optimum thermal condition for developing Thai BG with high SAC content base on methods A (70 °C and 80% RH) and B (60–75 °C and 80–85% RH). The total phenolic contents and antioxidant activities of Thai fresh garlic, Thai BG, and their powder forms were also compared. Method A worked the best for both types of garlic. The results indicated that the SAC content increased significantly after 7 days of fermentation and decreased drastically afterward with prolonged heat treatment. The optimum thermal condition for producing Thai fresh garlic and Chinese fresh garlic with high SAC content was 70 °C and a relative humidity of 80% for 12 days in an industrial fermentation chamber. The SAC content of Thai BG and Chinese BG increased approximately 139- and 122-fold, respectively. Furthermore, significant antioxidant capabilities determined by ferric ion-reducing antioxidant power, 2,2-diphenyl-1-picrylhydrazyl radical scavenging, and oxygen radical absorbance capacity assays were 34-, 6-, and 3-fold higher, respectively, than those of fresh garlic.

## 1. Introduction

To increase the health benefits of garlic (*Allium sativum* L.), black garlic (BG) products with a unique pleasant flavor and gelatin texture are currently popular in Thailand and other Asian countries [1,2,3]. BG is produced from raw garlic that has been processed at temperatures ranging from 30 to 90 °C with 50–90% relative humidity (RH) for 10–90 days without any additives [3,4,5,6,7]. This thermal treatment is called an aging process [8], where fresh white garlic is turned into BG with a soft or jelly-like texture and sweet-sour taste during the process [8]. In addition, a study reported that the quality and pleasant flavor of BG depended on fermentation processes (controlling temperature, RH, and fermented time) and garlic quality (species, planting area, climate, growth condition, etc.) [9]. Regarding its healthy properties, BG is effective in decreasing oxidative stress and inflammation and in reducing the risk of diabetes, hypertension, hypercholesterolemia, hyperlipidemia, arteriosclerosis, and cancer [3,9,10]. These health-related properties of BG are linked to its bioactive components such as S-allyl cysteine (SAC), S-allyl mercaptocysteine, S-1-propenylcysteine, diallyl sulfide, diallyl disulfide, diallyl trisulfide, polyphenols, alkaloids, and products of other Maillard reactions [11,12].

SAC is one of the main characteristic compounds in BG, which can be used as an evaluation index for the preparation of high-quality BG [5,13]. This SAC is synthesized through hydrolysis of γ-glutamyl bond in γ-L-glutamyl-S-allyl-L-cysteine (GSAC) by γ-glutamyl transpeptidase (GGT) [8,14,15,16,17,18]. GSAC is mainly deglutamylated prior to S-oxygenation in alliin biosynthesis in garlic, and GGT catalyzes transpeptidation more efficiently than hydrolysis [16]. Moreover, high SAC contents led to elevated antioxidant activities in BG [2,19]. Since the optimal GGT activity was achieved at 70 °C [17] and a processing RH of 80% [7], BG produced from Vietnam fresh garlic, Phan Rang, at 70 °C and 80% RH for 38 days of fermentation exhibited SAC content increasing from 3.5 µg/g to 159.81 µg/g (46-fold increase) [20]. Under these conditions, the polyphenol content of BG increased by sevenfold, whereas the 2,2-diphenyl-1-picrylhydrazyl (DPPH) radical scavenging activity increased 22-fold [20]. Even though optimal GGT conditions were reported, an increase in SAC content in BG was based on various temperatures during fermentation processes [2]. The SAC content in Korean garlic heated at 40 °C in 70% RH and fermented for 45 days increased 6.36-fold compared with that in fresh garlic, which was higher than the one heated at 70 °C (5.78-fold increase compared with fresh garlic) [2]. According to these studies, the mechanism underlying an increase in SAC content during BG production remains unclear.

In Thailand, commercially available BG is prescribed as ready-to-eat food and must meet the SAC requirement of >160 mg/kg fresh weight [21]. The industrial sector tends to request increasing the daily amount of BG consumption, whereas both indirect and direct BG consumption forms based on the active ingredient of various foods. This BG product is produced from big-head (single clove or monobulb) garlic, which is preferred by consumers in both households and the food industry because of its ease of consumption. Although many studies have focused on the fermentation steps and health benefits of BG produced from single-clove garlic, no data on the optimum aging process of BG with high SAC content from multi-clove garlic is available. This multi-clove garlic is the main economical local crop of Thailand, whereas single-clove garlic is an imported product as it can only be produced on a limited basis in Thailand because of an unsuitable climate [22]. Moreover, a mechanism for an increase in SAC content in BG is unclear, whereas information on other bioactive compounds (such as phenolics) and the health benefits (such as antioxidant activities) of multi-clove BG is limited. Therefore, to increase the utilization and commercial value of multi-clove garlic, this study aimed to investigate the optimum thermal conditions for the development of BG from multi-clove garlic with SAC content higher than that in single-clove garlic. Furthermore, the total phenolic contents (TPCs), total flavonoid contents (TFCs), and antioxidant activities of fresh multi-clove garlic, multi-clove BG, and their powder forms were investigated. Phenolic profiles based on liquid chromatography-electrospray ionization-tandem mass spectrometry (LC-ESI-MS/MS) of multi-clove fresh garlic, its BG, and powder forms were also determined. Knowledge gained from this study would support BG and its powder form as a potential functional food.

## 2. Materials and Methods

### 2.1. Sample Selection, Preparation, and Extraction

Multi-clove garlic was purchased from an organic farm, which was cultivated in the Community Enterprise Thalai Sub District Community Sufficiency Economy Learning Center Cultivated, Kanthararom District, Si Sa Ket Province, Thailand. The Thai government has added garlic from Si Sa Ket Province to the country’s geographical indication listed on 18 June 2020. Si Sa Ket garlic has a distinctive and unique smell with a different taste from garlic grown in other locations. Multi-clove garlic samples at day 63 of the planting stage, cultivated from January to March 2020, were cultivated for heat treatment, and samples at day 71 after planting between December 2020 and February 2021 were cultivated for making BG and its powder form. Chinese garlic (single clove) was purchased from Talaad-Thai, the wholesale market for agricultural products in Thailand. They were cleaned, damaged parts removed, transported to the laboratory, and kept dry at room temperature for the experiment. After reaching the optimum condition, bulb morphology, such as the number of cloves and the size and weight of garlic samples for making BG and its powder form, was recorded, and moisture content was measured [23].

The morphological characteristics of multi-clove garlic cultivated from Si Sa Ket Province (A) and single-clove garlic (B) purchased from Talaad-Thai are shown in Supplementary Figure S1 and Table S1.

BGs were produced in a BG fermentation chamber (size 1.50 m × 1.43 m × 1.85 m, 4.5 kilowatts, 380 volts, capacity 100 kg) with a temperature and humidity-programmable controller (TEMI 580, Maria Agricultural Machinery Stock Co., Ltd., Jining, China). A batch of each 7 kg of Thai garlic and Chinese garlic was produced without removing the outer layer using heat treatment methods A and B. For method A, the temperature was set to 70 °C and RH at 80% and maintained for 42 days, whereas method B was set in a stepwise heating schedule: (1) 75 °C at 85% RH for 9 days, (2) 70 °C at 80% RH for 12 days, and (3) 60 °C at 80% RH for 21 days by opening the chamber to collect the samples for measuring the moisture and SAC content every week.

After the analysis of SAC content, method A (at 70 °C and 80% RH) was selected for producing BG from 150 kg of Thai garlic and 100 kg of Chinese garlic in the industrial scale chamber specific for BG fermentation (size 4.50 m × 2.25 m × 2.1 m, 8 kilowatts, 380 volts, capacity 1800 kg) with the temperature and humidity-programmable controller model TEMI 580 (400-836-1617, OHSAS 18001:2007, Maria Agricultural Machinery Stock Co., Ltd., Jining, China). Both BG samples were maintained in the chamber for 12 days at 70 °C and 80% RH. The chamber was turned off once the color became black; the texture was tender and soft, almost jelly-like, and it had a slightly sweet taste. After turning off the chamber, the BGs were left inside overnight while the chamber’s door was slightly opened to gradually decrease the temperature, improve its sweet taste, and dry the surface. The BG was kept at 4 °C, and samples were collected for the measurement of the moisture and SAC contents. To prepare BG powder, Thai BGs were peeled off and then placed in a hot air oven at 60 °C for 41 h, and samples were collected.

Thai fresh garlic, Thai BG, and their powder forms were extracted according to the method described by Thach and Thuy [24], with slight modifications in which an ultrasonic bath was used to enhance the mass transfer of the solvent into the plant material [25,26]. Furthermore, the samples were weighed at 1 g dry weight (DW), added into 10 mL of 50% (*v*/*v*) ethanol in a 50-mL Conical bottom centrifuge tube, vortexed for 1 min, and sonicated at 60 °C for 90 min in an ultrasonic bath (CCA-1111-CE, Shanghai Eyela C. LTD, Shanghai, China) The supernatant was collected after centrifugation at 3500 rpm for 20 min. Furthermore, Thai fresh garlic, Thai BG, and their powder forms were extracted using various solvents, i.e., 70% *(v/v)* methanol, water, and ethyl acetate, and sonicated for 10 min at 70 °C following the method by Theppakorn and Wongsakul [27]. The sample extract was stored at −20 °C in the storage vials to determine the TPCs, TFCs, phenolic profiles by LC-ESI-MS/MS, and their antioxidant properties.

### 2.2. Measurement of Moisture Content

According to the methods proposed by the Association of Official Agricultural Chemists 2000 (AOAC 2000) [28], 4 g of samples were weight in the dish of the moisture analyzer (HE53, Mettler-Toledo, Greifensee, Switzerland) and heated at 105 °C. During heat treatment, the moisture content of all fresh garlic, BG, and their powder forms was determined, and the color was observed every week.

### 2.3. Determination of SAC

SAC was analyzed at the Department of Chemistry, Faculty of Science, Burapha University, by high-performance liquid chromatography using Agilent 1260 Infinity II (Ag Technologies, Inc., CA, USA) with photodiode array detector (DAD, λ = 250 nm, injection volume = 10 µL) according to the method described by Malapong et al. [29]; 1 g of chopped BG in 15 mL of distilled water was derivatized with dansyl chloride at room temperature for 15 min at a mobile phase of 45% (*w*/*v*) sodium acetate buffer, pH 5, and 55% (*v*/*v*) methanol in a Poroshell C-18 column. The amount of SAC was calculated from the peak area of the chromatography [29].

### 2.4. Determination of TPCs, TFCs, and Phenolic Profiles by LC-ESI-MS/MS

#### 2.4.1. Determination of TPCs

The TPCs of the sample extracts were determined using the Folin–Ciocalteu reagent following the modified methods of Amarowicz et al. [30], Swain and Hillis [31], and Naczk and Shahidi [32]. The extracts of Thai fresh garlic, Thai BG, and their powder forms were diluted, and 10 µL of each extract was transferred into a 96-well microplate containing 160 µL of distilled water. Furthermore, 10 µL of Folin–Ciocalteu reagent and 20 µL of the 20% (*w*/*v*) sodium carbonate solution were added, mixed well, incubated at room temperature for 30 min, and protected from light. Absorbance was measured at a wavelength of λ = 750 nm using a microplate reader (SN 13120913, Bio Tek Instruments Inc., Winooski, VT, USA). Gallic acid (0–250 μg/mL) was used as the standard. The results were expressed as mg gallic acid equivalent (GAE)/g DW after taking into account the applied dilution schemes and calculations based on the calibration curve for gallic acid (y = 0.0028x + 0.0067, R2 = 0.9985). The determinations were performed in five independent replications.

#### 2.4.2. Determination of TFCs

The TFCs of the aqueous ethanolic extract from Section 2.1 were investigated using a well-established protocol, as previously reported [33]. This colorimetric assay employed aluminum chloride as the assay reagent and quercetin (0–100 µg/mL) as the standard. The assay was monitored on a SynergyTM HT 96-well UV-visible microplate reader and Gen5 Data Analysis Software (Bio Tek Instruments, Inc.). The result was expressed as mg quercetin equivalent (QE)/g DW after taking into account the applied dilution schemes and calculations based on the calibration curve for quercetin (y = 0.0026x + 0.0043, R2 = 0.9890). The determinations were performed in five independent replications.

#### 2.4.3. Determination of Phenolic Profiles

Multi-clove fresh garlic, its BG, and powder forms were analyzed regarding their phenolic profiles by LC-ESI-MS/MS according to a well-established protocol and validation as previously reported [34]. The LC–ESI-MS/MS system consisted of a Dionex Ultimate 3000 series ultrahigh-performance liquid chromatography, a DAD with a Chromeleon 7 chromatography data system (version 7.2.9.11323), a TSQ Quantis Triple Quadrupole mass spectrometer, a 2.1 mm × 100 mm, and a 2.6-μm Accucore RP-MS Column (Thermo Fisher Scientific, Bremen, Germany). A gradient system of acetonitrile (solvent A) and 0.1% (*v*/*v*) formic acid in Milli-Q water (18.2 MΩ·cm resistivity at 25 °C, solvent B) with a flow rate of 0.5 mL/min was set up as follows: 10–80% solvent A and 90–20% solvent B at 0.0–8.0 min; 80–10% solvent A and 20–90% solvent B at 8.0–8.1 min; 10% solvent A and 90% solvent B at 8.1–10.0 min. The standards included 27 purchased phenolics, namely, apigenin (>98.0% HPLC), hesperidin (>90.0% HPLC, T), (−)-epigallocatechin gallate (>98.0% HPLC), chlorogenic acid (>98.0% HPLC, T), 3,4-dihydroxybenzoic acid (≥97% T), p-coumaric acid (>98.0% GC, T), 4-hydroxybenzoic acid (>99.0% GC, T), caffeic acid (>98.0% HPLC, T), genistein (>98.0% HPLC), luteolin (>98.0% HPLC), myricetin (>97.0% HPLC), kaempferol (>97.0% HPLC), syringic acid (>97.0% T), cinnamic acid (>98.0% HPLC), ferulic acid (>98.0% GC, T), naringenin (>93.0% HPLC, T), sinapic acid (>99.0% GC, T), and quercetin (>98.0% HPLC, E) from Tokyo Chemical Industry (Tokyo, Japan); epicatechin (≥98% HPLC), catechin (≥98% HPLC), epicatechin gallate (≥98% HPLC), vanillic acid (≥97% HPLC), rutin (≥94% HPLC), rosmarinic acid (≥98% HPLC), and gallic acid (97.5–102.5% T) from Sigma-Aldrich (St. Louis, MO, USA); isorhamnetin (≥99.0% HPLC) from Extrasynthese (Genay, France); and galangin (≥98.0% HPLC) from Wuhan ChemFaces Biochemical Co., Ltd. (Wuhan, China).

### 2.5. Determination of Antioxidant Activities

The ferric ion-reducing antioxidant power (FRAP) and DPPH radical scavenging assays were employed according to established methods [35]. The oxygen radical absorbance capacity (ORAC) assay was performed based on the method of Ou et al. [36]. Spectrophotometrically, antioxidant activities were measured using a SynergyTM HT 96-well UV-visible microplate reader and a Gen5 Data Analysis Software (Bio Tek Instruments, Inc., Winooski, VT, USA). Trolox served as the benchmark standard, and the results were expressed as mM Trolox equivalent (TE)/g DW. All chemicals and reagents were purchased from Sigma-Aldrich.

### 2.6. Statistical Analysis

All experiments were conducted in triplicate to pentaplicate measurements of 3–5 independent sets of samples (n = 3–5). The results were represented as a mean ± standard deviation. A paired samples test and one-way analysis of variance with Tukey’s post-hoc test were performed, with significant differences at *p* < 0.05. Statistical analysis was performed using IBM SPSS Statistics for Windows, version 19.0 (IBM Corp., Armonk, NY, USA).

## 3. Results

### 3.1. Effect of Different Thermal Processes on Moisture Content

**Figure 1:**
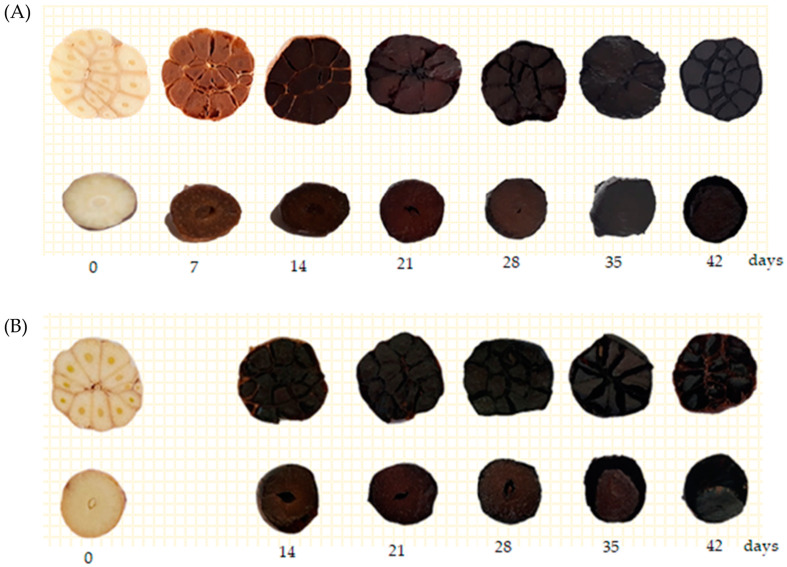
Changes in the color of Thai fresh garlic strain and Chinese garlic strain during period of thermal processes methods; (**A**) temperature was set at 70 °C, 80% RH, 42 days and (**B**) temperature was set at Step 1: 75 °C, 85% RH for 9 days. Step 2: 70 °C, 80% RH for 1 days; Step 3: 60 °C, 80% RH for 21 days.

For heat treatment, the moisture content of Thai garlic cultivated from January to March was 67.30–68.14%, whereas that of Chinese garlic was 58.04–58.63%. During method A, the water evaporation rates until day 41 were 58.26 and 56.25% in Thai and Chinese garlic samples, respectively, and the moisture contents of Thai and Chinese fresh garlic samples in method B were 14.22 and 15.39%, respectively. At the end of the aging process on day 42, the moisture contents of both BGs from method B were lower than those of method A. Changes in the color of the Thai and Chinese garlic samples during methods A and B are shown in Figure 1.

### 3.2. Effect of Different Thermal Processes on SAC Content

Changes in SAC content after methods A and B in each Thai and Chinese garlic sample using the BG fermentation chamber are shown in Table 1. The SAC content rapidly increased at the initial step and then decreased significantly when the heat treatment progressed. The Thai BG samples subjected to method A had significantly higher SAC content than Chinese BG samples, whereas Chinese BG samples subjected to method B had significantly higher SAC content than Thai BG samples.

The SAC content of Thai BG was still higher than that of Chinese BG under optimum thermal conditions of 70 °C and 80% RH in both BG fermentation chambers. Both BG samples were maintained in the BG fermentation chamber for only 12 days to achieve a suitable texture; the color characteristics of the fresh garlic, Thai BG, and Chinese BG were observed, and their morphological characteristics are shown in Table 2. The moisture content and weight of Thai and Chinese BG cloves without shells decreased by half compared with fresh garlic, whereas the SAC content increased approximately 139-fold and 122-fold compared with those of fresh garlic, respectively. However, the SAC contents of Thai and Chinese BG samples in different fermentation chambers and heat treatment times are shown in Table 3, and no significant difference was found (*p* = 0.68 and 0.05, respectively).

To make BG powder, the selected Thai BG samples were kept at 4 °C and dried in a hot air oven at 60 °C for 41 h. The stability of SAC in Thai BG and its powder form between storage times of 2 days and 162 days is shown in Supplementary Table S2. The moisture content of Thai BG decreased from 32.53 to 27.47%. In addition, the SAC content significantly decreased from 781.98 ± 17.23 to 667.51 ± 10.30 mg/kg DW, which accounted for 14.64%. The moisture content of Thai BG powder decreased from 7.99 to 6.28% and its SAC content significantly decreased by 12.53% from 626.52 ± 9.20 to 547.99 ± 3.87 mg/kg DW. However, the SAC content of Thai BG powder decreased from their fresh weight by 19.88% and 17.91% at a storage time of 2 days and 162 days, respectively.

### 3.3. Effect of Heat Treatment on SAC Content, Total Polyphenol Contents, TFCs, and Phenolic Profiles of Thai Garlic (Fresh, BG, and Their Powder Forms) Analyzed by LC-ESI-MS/MS

Thai BG subjected to thermal aging at 70 °C and 80% RH and maintained in the fermentation chamber for 12 days had significantly higher SAC content than its powder form (*p* < 0.05), whereas the total polyphenol contents (mg gallic acid/g dry weight) and TFCs of Thai BG powder were the highest than those of Thai fresh garlic and Thai BG (Table 4).

The total polyphenol contents in the 50% *(v/v)* ethanol extracts of Thai BG powder samples were between 10.58 ± 0.50 and 10.84 ± 0.20 mg gallic acid/g extract, whereas when extracted with 80% *(v/v)* methanol, the TPCs were at 9.09 ± 0.41 mg gallic acid/g extract (Supplementary Table S3). Extracting a 1 g DW sample with 10 mL of 50% (*v*/*v*) ethanol sonicated for 90 min at 60 °C resulted in an extract yield of 68%, which was selected for sample preparation and the determination of phenolic profiles.

The 24 phenolic compounds were not found in the LC-ESI-MS/MS analysis of Thai fresh garlic, Thai BG, or their powder extracts at various concentrations up to 700 mg extract/mL after acid hydrolysis reaction. In addition, none of the four samples contained epicatechin, catechin, or epicatechin gallate after extraction in a mixture of 70% (*v*/*v*) methanol, water, and ethyl acetate for 10 min at 70 °C (Supplementary Figure S2).

### 3.4. Effect of Heat Treatment on the Antioxidant Capacities of Thai Garlic Samples (Fresh, BG, and Their Powder Forms)

For Thai BG subjected to a heat treatment at 70 °C and 80% RH and maintained in a large-scale fermentation chamber for 12 days, the antioxidant activities (DPPH and ORAC) of Thai BG powder were significantly higher than those of Thai fresh garlic and Thai BG, whereas the antioxidant activity (FRAP) of Thai BG powder was significantly higher than that of Thai fresh garlic (Table 4). The radical scavenging activity or the DPPH scavenging effect (%) was expressed as the inhibition concentration (IC50; Supplementary Figure S3). The IC50 of vitamin C (positive control), Thai BG, and its powder forms were 5.56, 713.23, and 491.26 µg/mL, respectively.

## 4. Discussion

An increasing SAC content is an important change that occurs during the heat treatment for BG production. The end quality of BG depends on critical factors such as processing temperature, humidity, processing time, cultivar, and types and characteristics of fresh garlic [8,37].

Garlic is one of the most economically significant crops in Thailand. More than 90% of the garlic plantations in Thailand are in the northern region [38]. Sunanta et al. (2020) reported morphological characteristics of Thai garlic (multi-clove) samples grown between November and March in the northern provinces of Thailand (i.e., Chiangmai, Lamphun, Lampang, Chiagrai, and Maehongson), and their bulb weight, clove number, and clove weight were between 6.15 and 15.49 g, 9–19, and 0.59–0.94 g, respectively [23]. Supplementary Table S1 shows the raw materials for making BG and the morphological features of fresh garlic from Si Sa Ket Province, which is grown between December and February in the northwest of Thailand, were 9.66–19.86 g/bulb, 9–21 cloves/bulb, and 0.49–1.12 g/clove, respectively. Nevertheless, the mean number of cloves and bulb weight of Thai fresh garlic (multi-clove) from Si Sa Ket Province were similar to those of Thai fresh garlic (multi-clove) from Chiagrai and Chiangmai provinces. A garlic bulb typically contains approximately 20 cloves; however, the clove number per bulb, weight per clove, organic sulfur content, and nutrient composition varied between cultivars, growing season, soil type, environment, crop growth conditions, and planting location [8,39]. Likewise, the clove weight of Chinese garlic samples was 8.6 times higher than that of Thai fresh garlic (multi-clove), which influenced the optimum thermal condition for producing quality BG.

The water content of BG is an important parameter that influences the texture of BG [2]. In this study, differences were noted in the initial moisture content between Thai garlic and Chinese garlic. Chinese garlic samples, which were imported from China, had a lower moisture content than Thai garlic. Moreover, the initial moisture content differed in the processing times of methods A and B. This result revealed that temperature affects the color of BG, changing from white to dark brown, and reduces the moisture content of the samples. Methods A and B induced changes in the color of Thai fresh garlic and Chinese garlic samples. The blackness of BG does not mean that the BG had better quality [6]. The moisture content reduced faster in harder BG produced at higher temperatures (75 °C, 70 °C, to 60 °C) and 80–85% RH for 42 days in method B. Bae et al. (2014) and Zhang et al. (2016) also found that lower moisture contents were noted at higher temperatures, which results in bone-dry and harder BG samples [2,6]. Zhang et al. (2016) found that soft and wet BG had a moisture content of 50–70% at the early stage of BG processing; however, harder BG contains <35% of moisture. The garlic cell is dried off when subjected to a dehydrating process. Furthermore, the pectic polysaccharides of the primary cell wall are destroyed by heat treatment; as a result, BG becomes softer than fresh garlic [6,40]. Sila et al. (2006) also suggested that heat treatment causes a pronounced degradation of the pectic polysaccharides in vegetables and fruits, resulting in reduced intercellular adhesion and consequently increased softening [41].

SAC is an important bioactive compound of quality BG samples. Different heat treatment methods and conditions can change the components of fresh garlic into various compounds [5,13,42]. To the best of our knowledge, this is the first study investigating SAC content during methods A and B in Thai BG and Chinese BG samples. Moreover, optimum processing conditions were repeated in fermentation chambers of different sizes to determine the SAC content from the same cultivar, planting area, and type of fresh garlic, including different batches of plantation. In addition to clarifying changes in the SAC content of BG products, we investigated the SAC content of Thai BG powder forms, which had a high SAC content under optimum conditions, and compared the stability of SAC during storage. Our results show that Thai BG had the highest SAC content when processed under method A for 7 days; however, the color and texture were not yet suitable for consumption when compared with commercial BG. The SAC content of Thai BG was significantly higher when the garlic was exposed to lower temperatures. The Thai BG processed under method A (70 °C and 80% RH for 14 days) should be the optimum condition for the production of Thai BG with high SAC content. Based on SAC contents, the optimum heat treatment time should not be more than 14 days; however, it was dependent on the moisture of the raw materials and BG products during heat treatment. Methods A and B could produce Chinese BG with a high SAC content after <14 days of heat treatment. Thus, methods process A (70 °C and 80% RH for 12 days) and B (60–75 °C and 80–85% RH within 14 days) could be used for the production of Chinese fresh garlic (single clove). However, the heat treatment time depends on the general characteristics of BG, such as moisture, texture, color, and taste.

Clearly, the SAC content increases significantly compared with that of fresh garlic after 7 days of fermentation at 70 °C and 80% RH and decreases significantly with increased thermal treatment. These results agree with those of Molina-Calle et al. (2017), i.e., when Rocambole fresh garlic was treated at 60–80 °C with high humidity for 36 days. GSAC, the substrate of SAC, was highly concentrated in fresh garlic, but its concentration decreased along with the heat treatment; thus, a good correlation was found, i.e., the SAC content was the highest at the earlier period of heat treatment and the lowest with extended treatment [43]. Moreover, Liu et al. (2022) found that GGT showed a downward trend during BG processing [7]. On the contrary, Bae et al. (2014) reported different results, demonstrating that the SAC content in Korean BG subjected to various temperatures 40 °C, 55 °C, 70 °C, and 85 °C) and 70% RH for 45 days increased significantly as the heat treatment progressed [2]. Despite the abundance of water, GSAC was transformed into SAC [44].

When comparing fermentation chambers of different sizes under the same treatment process (70 °C and 80% RH), the SAC contents of Thai BG and Chinese BG were not significantly different; however, the SAC content of Thai BG was significantly higher than that of Chinese BG under optimum thermal conditions (70 °C and 80% RH) if the treatment time in the fermentation chamber differed by 2 days. We found that the weight of Thai BG and Chinese BG cloves decreased when compared with that of fresh garlic by approximately 52% and 47%, and the SAC content increased by approximately 139 and 122 times when compared with that of fresh garlic, respectively. While Quan et al. (2020) reported 12 days, Vietnam BG placed in the cooker at 70 °C with 70–80% RH had a SAC content of 663.6 mg/kg DW, or 12 times that of fresh garlic [45]. The content requirements of SAC in Thai BG shall not be less than 160 mg/kg fresh weight [21]. However, the SAC contents of our Thai BG and Chinese BG were higher than the Thai FDA requirement of 3.29 and 2.91 times, respectively.

This study observed that the moisture content of Thai BG decreased from 32.53 to 27.47% in 2 days and 162 days at 4 °C after heat treatment of BG. Interestingly, the stability of SAC in Thai BG and Thai BG powder during storage significantly decreased by 14.64% and 12.53%, respectively. Thus, an oxidation process is responsible for the deterioration of the quality of food products. SAC is oxidized to alliin by S-oxygenation [16]. In addition, Fitrotin et al. (2019) revealed that the antioxidant activities of BG during storage in a refrigerator decreased by 34.61%, whereas it decreased by 81.13% during storage at room temperature for 3 months [46]. The rates of Millard reaction and oxidation of highly active compounds such as SAC, polyphenol, and unsaturated fatty acids depend on the storage temperature and environmental temperature [8].

In this study, SAC (mg/g DW), total polyphenol contents (mg gallic acid/g dry weight), and TFCs (mg QE/g DW) of Thai BG increased 176-fold, 11-fold, and 2-fold from those of Thai fresh garlic, respectively.

Although the results of this study revealed that the phenolic profiles of Thai fresh garlic, Thai BG, and their powder forms were not detected, despite the use of various extraction conditions and 27 authentic standards of phenolics by LC-ESI-MS/MS, the TPCs of the samples were less than the limit of detection according to Sirichai et al. (2022) [34]. Meanwhile, Najman et al. (2020) detected total polyphenols of 12.50 mg GAE/g DW and phenolics such as quercetin, p-coumaric acid, cholorogenic acid, gallic acid, catechin, gallate epigallocatechin, and epicatechin from Poland BG treated at 70 °C and 80% RH for 45 days by HPLC UV/VIS detector [47]. The results varied because different fermentation times were employed. Our heat treatment process involved treating Thai fresh garlic at 70 °C and 80% RH and storing the samples in the fermentation chamber for 12 days; the total polyphenol contents detected were approximately two times less than those reported by Najman et al. (2020) (45 days of fermentation). This information supports our finding that we could not detect phenolic profiles by LC-ESI-MS/MS. Considering that different temperatures during fermentation may affect the total polyphenol content, Kim et al. (2013) also reported total phenolic profiles such as epicatechin, gallocatechin, epigallocatechin, and quercetin by HPLC in Korea for BG treated at 60–90 °C and 60–100% RH for 14 days, whereas our samples were fermented at 70 °C and 80% RH for 12 days. There is a definite possibility that the heating process of BG at higher temperatures improves the phenolic content due to the cleaving of bound forms such as esterified and glycosylated forms, leading to an increase in free forms [48]. Moreover, Zhang et al. (2016) reported that the total polyphenol content significantly increased during thermal treatment [6].

SAC is a major organosulfur molecule that contains a thiol group (-SH functional group) that prevents lipid or protein oxidation and nitration by donating its hydrogen atom or proton (H+) to an electrophilic species [49]. Thiols are powerful antioxidants that can act as metal chelators, electron acceptors, radical quenchers, and substrates for specific redox reactions, reducing unstable free radicals by oxidizing processes [50,51], whereas polyphenols can neutralize free radicals by donating an electron or hydrogen atom [52]. A good correlation was found between the antioxidant capacities (FRAP, DPPH, and ORAC) of Thai BG, which were approximately increased compared with those of Thai fresh garlic (multi-clove) at 97%, 84%, and 65%, respectively. Similar to the results of Thai garlic grown in northern Thailand, Sunanta et al. (2020) reported that the DPPH radical scavenging activity, total polyphenol contents (mg gallic acid/g dry weight), and TFCs (mg catechin/g DW) of Thai BG (multi-clove) increased by 86%, i.e., 7–13-fold and 3–5-fold from those of Thai fresh garlic (multi-clove), respectively [23].

This study also showed that the antioxidant capacities (DPPH and ORAC) of Thai BG powder significantly increased after the treatment of Thai BG at 60 °C for 41 h in a hot air oven. This result may be due to the increased total polyphenol content (mg gallic acid/g DW) and TFCs (mg quercetin/g DW) in Thai BG, at levels of 13% and 47%, respectively. Several studies have explained that heat treatment can separate the bound polyphenols and flavonoids from the cell wall [4,52,53], but it decreased the SAC content of Thai BG by 18%.

According to Colin-Gozalez et al. (2012), the allyl group of SAC is necessary to preserve scavenging activities, such as scavenging superoxide anion, hydrogen peroxide, hydroxyl radical, and peroxynitrite anion [49]. This study examined the DPPH scavenging effect (%) that was expressed as the inhibition concentration (IC50) of vitamin C (positive control), Thai BG, and its powder forms, with 5.56, 713.23, and 491.26 µg/mL, respectively. Other studies have also reported that the IC50 of vitamin C ranged from 4.57 to 6.10 µg/mL [54,55,56]. Azizah et al. (2020) demonstrated that the IC50 of Indonesian BG treated at 70–90 °C for 12 days in a rice cooker was 637.80 µg/mL [57]. These results confirm that different factors, heat treatments, and sources of raw materials (i.e., plantation area, environment, season, etc.) can have different results.

## 5. Conclusions

SAC is the most abundant bioactive compound in BG. To produce Thai fresh garlic (multi-clove) and Chinese fresh garlic (single clove) with high SAC contents, the optimum treatment condition was found to be 70 °C and 80% RH for 12 days in an industrial fermentation chamber. The SAC contents of our Thai BG and Chinese BG increased approximately 139 and 122 times, respectively, when compared with that of fresh garlic. Although the phenolic profile of Thai BG could not be detected, Thai BG still showed high antioxidant capacities as determined by FRAP, DPPH, and ORAC, which were higher than those of fresh garlic by 34, 6, and 3 times, respectively. Thus, factors that should be considered for BG production were the heat treatment condition, type and characteristics of the raw materials, and general characteristics of BG (i.e., moisture, texture, color, and flavor). However, further studies should confirm their functional and health-related benefits in a clinical study.

## Figures and Tables

**Table 1:** Changing of SAC content after treated with thermal process method A and B in Thai garlic strain and Chinese garlic strain.

Time (Days)	RTGA	RCGA	RTGB	RCGB
0	193.39 ± 1.91 ^aA^	234.69 ± 24.39 ^aA^	ND	ND
7	1563.18 ± 12.10 ^bA^	1008.80 ± 34.42 ^bB^	-	-
14	802.56 ± 40.38 ^cA^	568.16 ± 21.36 ^cB^	393.67 ± 22.74 ^aC^	518.84 ± 8.40 ^aB^
21	424.62 ± 22.95 ^dA^	355.81 ± 37.03 ^dA^	204.19 ± 6.22 ^bB^	406.55 ± 5.62 ^bA^
28	233.18 ± 11.50 ^aA^	321.84 ± 14.32 ^dB^	182.72 ± 10.51 ^cC^	303.67 ± 15.43 ^cB^
35	117.57 ± 10.78 ^eA^	190.67 ± 19.95 ^aeB^	ND	221.86 ± 10.49 ^dBC^
42	105.82 ± 16.27 ^eA^	112.19 ± 3.81 ^fA^	ND	107.22 ± 8.32 ^eA^

Results are presented as mean ± standard deviation in mg/kg DW; RTGA: Thai fresh garlic strain (multi clove) treated with thermal process method A; RCGA: Chinese garlic strain (single clove) treated with thermal process method A; RTGB: Thai fresh garlic strain (multi clove) treated with thermal process method; RCGB: Chinese garlic strain (single clove) treated with thermal process method B; Different letters (a–f) in each column indicate significant differences at *p* < 0.05 by Tukey’s Honestly Significant Difference Test; Different capital letters (A–C) in each row indicate significant differences at *p* < 0.05 by Tukey’s Honestly Significant Difference Test; ND= not detected, limit of detection (LOD) = 3.8 mg/kg, N = 3.

**Table 2:** The characteristics data of fresh garlic, Thai BG and Chinese BG when treated with the thermal process at 70 °C, 80% RH by maintained in black garlic fermentation chamber for 12 days.

	Thai Fresh Garlic (Multi Clove)	Thai BG (Multi Clove)	Chinese Fresh Garlic (Single Clove)	Chinese BG (Single Clove)
**Morphological appearances**	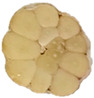	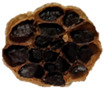	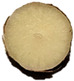	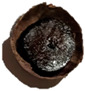
**Moisture** ** (%) **	64.31 ± 0.72	32.53 ± 0.50	58.00 ± 0.26	28.94 ± 0.53
**SAC** ** ( ** **mg** ** / ** **kg fresh wt** ** ) **	ND	527.57 ± 11.63	ND	464.80 ± 8.98
**SAC** ** ( ** **mg** ** / ** **kg DW** ** ) **	ND	781.98 ± 17.23	ND	654.29 ± 12.64
**Weight of clove** ** ( ** **g** ** ) **	0.77 ± 0.18	0.37 ± 0.06	6.63 ± 1.03	3.49 ± 0.62

Note: Results are presented as mean ± standard deviation in mg/kg; ND = not detected, LOD = 3.8 mg/kg, N = 3.

**Table 3:** The SAC content of Thai BG, and Chinese BG in different fermentation chamber and different time of thermal process in test process (14 days) and run process (12 days) of fermentation periods at 70 °C, 80% RH.

Size of Chamber and Fermentation Periods	SAC Content (mg/kg Dry Weight)	
	Thai BG	Chinese BG
Test process: small scale chamber/14 days	802.56 ± 40.38 ^aA^	568.16 ± 21.36 ^aB^
Run process: large scale chamber/12 days	781.98 ± 17.23 ^aA^	654.29 ± 12.64 ^aB^

Results are presented as mean ± standard deviation in mg/kg DW N = 3; Different letters (a–b) in each column indicate significant differences at *p* < 0.05 by Paired Samples Test; Different capital letters (A–B) in each row indicate significant differences at *p* < 0.05 by Paired Samples Test.

**Table 4:** Comparative SAC content, total polyphenols, total flavonoids, antioxidant activities of Thai strain garlic (fresh garlic, Thai BG and their powders) fermented in large scale chamber at 70 °C, 80% RH in 12 days.

	Thai Fresh Garlic	Thai Fresh Garlic Powder	Thai BG	Thai BG Powder
SAC contents	ND	ND	667.51 ± 10.30 ^A^	547.99 ± 3.87 ^B^
TPCs	0.47 ± 0.04 ^A^	0.99 ± 0.08 ^B^	5.28 ± 0.13 ^C^	6.04 ± 0.60 ^D^
TFCs	0.27 ± 0.02 ^A^	0.71 ± 0.03 ^B^	0.62 ± 0.05 ^C^	1.18 ± 0.04 ^D^
FRAP	0.66 ± 0.06 ^A^	2.65 ± 0.15 ^A^	22.11 ± 1.65 ^B^	23.05 ± 2.30 ^B^
DPPH	2.99 ± 0.25 ^A^	19.04 ± 1.51 ^B^	18.21 ± 1.20 ^BC^	21.28 ± 2.05 ^BD^
ORAC	42.96 ± 2.58 ^A^	139.38 ± 12.29 ^B^	121.10 ± 9.93 ^BC^	142. 46 ± 14.07 ^BD^

Note: Results are presented as mean ± standard deviation in mg/kg DW For SAC content: ND = not detected, LOD = 3.8 mg/kg, N = 3; For Total polyphenol, total Flavonoid, FRAP, DPPH and ORAC: N = 5; Different capital letters (A–D) in each row indicate significant differences at *p* < 0.05 by Tukey’s Honestly Significant Difference Test; Data shown in this table has already corrected % moisture at the same level; SAC content (mg/kg DW), Total polyphenol (mg gallic acid/g DW), Total Flavonoid (mg quercetin/g DW), FRAP (mM Trolox equivalent/g DW); DPPH (mM Trolox equivalent/g dry weight), ORAC (mM Trolox equivalent/g DW).

## Data Availability

Data are contained within this article and Supplementary Materials.

## Supplementary Materials

The following supporting information can be downloaded at: https://www.mdpi.com/article/10.3390/foods12061227/s1, Figure S1: Morphological appearances of Thai garlic (A); its clove cultivated from Si Sa Ket province (B) and Chinese garlic purchased from Talaad-Thai, the wholesale market for agricultural products of Thailand (C); Figure S2: Detection of phenolic profiles of epicatechin (1), catechin (2) and epicatechin gallate (3) in Thai fresh garlic (multi clove) (A), Thai fresh garlic (multi clove) powder (B), Thai BG (C) and Thai BG powder (D) by LC-ESI-MS/MS; Figure S3: The radical scavenging activity (% RSA) of Vitamin C (positive control) (A), Thai BG (B) and Thai BG powder (C); Table S1: The morphological characteristics of Thai garlic (multi clove) and Chinese garlic (single clove); Table S2: Stability of SAC contents in Thai BG and Thai BG powder; Table S3: Total polyphenol contents of Thai fresh garlic (multi clove) (A) and Thai BG powder (B) in difference extraction condition.
