# Supplementary material for: Effect of Thermal Processes on S-Allyl Cysteine Content in Black Garlic
Source: Foods. 2023 Mar 13;12(6):1227. doi: 10.3390/foods12061227 (PMC10048598; doi:10.3390/foods12061227)
Supplement: Supplementary file 1 [file foods-12-01227-s001.zip › foods-2238677-supplementary.pdf]

foods

Supplementary Materials

Effect of Thermal Processes on *S*-Allyl Cysteine Content in Black Garlic

Kanokwan Manoonphol [1], Uthaiwan Suttisansanee [2], Chadamas Promkum [2] and Chaniphun Butryee [2,*]

[1] Doctor of Philosophy Program in Nutrition, Faculty of Medicine Ramathibodi Hospital and Institute of Nutrition, Mahidol University, Bangkok 10400, Thailand
[2] Food and Nutrition Academic and Research Cluster, Institute of Nutrition, Mahidol University, Nakhon Pathom 73170, Thailand
* Correspondence: chaniphun.but@mahidol.ac.th, chaniphun25@gmail.com (C.B.); Tel.: +66-(0)-2800-2380 (ext. 119)

Supplementary Figure S1:

Thai garlic bulb from Si Sa Ket province (A and B) has thin skin, firm texture, spicy flavor with pungent and unique smell that make them distinctive with Chinese garlic strain (C). For the morphological data (size weight, number of cloves and weight) of Thai garlic and Chinese garlic for making BG and their powder after got optimum condition was shown in Supplementary Table S1. Thai garlic was round bulb with 9.66-19.86 g/bulb and circumference 26.02-35.76 mm. consisting of 9-21 individual cloves. On the other hand, Chinese garlic was round bulb with 4.37-8.34 g/bulb or individual clove and circumference 26.02-35.76 mm. The moisture of Thai garlic and Chinese garlic were 64.31% and 58%, respectively.

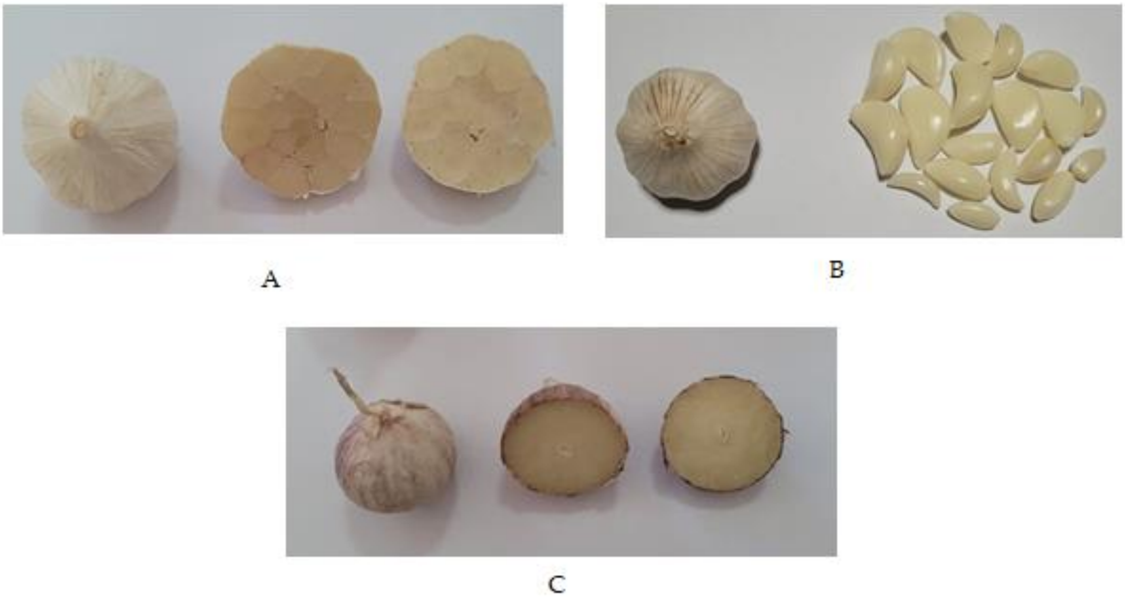

Figure S1. Morphological appearances of Thai garlic (A); its clove cultivated from Si Sa Ket province (B) and Chinese garlic purchased from Talaad-Thai, the wholesale market for agricultural products of Thailand (C).

Supplementary Table S1:

The morphological data of Thai garlic (multi clove) and Chinese garlic (single clove) samples

Table S1. The morphological characteristics of Thai garlic (multi clove) and Chinese garlic (single clove)

Type of garlic	Cultivated area	Cultivated duration	Bulb size (mm)	Clove number	Bulb weight [a] (g)	Clove weight [b] (g)
Thai fresh garlic	Kanthararom, Si Sa Ket	December - February	31.70 ± 2.42	15 ± 3	13.69 ± 1.63	0.77 ± 0.18
Chinese fresh garlic	China	No data	23.67 ± 1.25	1	6.92 ± 1.01	6.63 ± 1.03

Note: Results are presented as mean ± standard deviation; N of Thai garlic = 30 bulb, 436 cloves; N of Chinese garlic = 30 Cloves; [a] Bulb are weighed with shell in gram; [b] Clove are weighed without shell in gram.

Supplementary Table S2

The SAC content of Thai BG and Thai BG powder during storage times for 2 days and 162 days

Table S2. Stability of SAC contents in Thai BG and Thai BG powder

Storage times (days)	SAC content of Thai BG (mg/kg DW)	SAC content of Thai BG powder (mg/kg DW)
2	781.98 ± 17.23 [aA]	626.52 ± 9.20 [aB]
162	667.51 ± 10.30 [bA]	547.99 ± 3.87 [bB]

Note: Results are presented as mean ± standard deviation in mg/kg dry weight N = 3; Different letters (a-b) in each column indicate significant differences at $p < 0.05$ by Paired; Samples Test; Different capital letters (A-B) in each row indicate significant differences at $p < 0.05$ by Paired; Samples Test; Data showed in this table has already correct % moisture at the same level.

Supplementary Table S3

Extraction condition and % yield of Thai fresh garlic (multi clove) (A) and Thai BG powder (B)

Table S3. Total polyphenol contents of Thai fresh garlic (multi clove) (A) and Thai BG powder (B) in difference extraction condition.

Sample	Extraction condition	% yield	Total polyphenol (mg Gallic acid /g extract)
A	Ratio 1:4, 50 % *(v/v)* ethanol, sonicate at 60 °C 120 min	12.62	2.52 ± 0.17[a]
B	Ratio 1:4, 50 % *(v/v)* ethanol, sonicate at 60 °C 120 min	55.63	10.72 ± 0.25[b]
B	Ratio 1:4, 50 % *(v/v)* ethanol, sonicate at 60 °C 120 min	46.15	10.84 ± 0.20[b]
B	Ratio 1:10, 50 % *(v/v)* ethanol, sonicate at 60 °C 90 min	68.06	10.58 ± 0.50[b]
B	Ratio 1:8, 80 % *(v/v)* methanol, sonicate at 30 °C 10 min	47.40	9.09 ± 0.41[c]

Note: Ratio of Sample (g DW): solvent (mL); Results are presented as mean ± standard deviation, N = 3; Different letters (a-c) in each column indicate significant differences at $p < 0.05$ by Tukey's; Honestly Significant Difference Test.

Supplementary Figure S2:

Determination of epicatechin (1), catechin (2) and epicatechin gallate (3) in Thai fresh garlic (multi clove) and Thai BG (multi clove) and their powder (A-D)

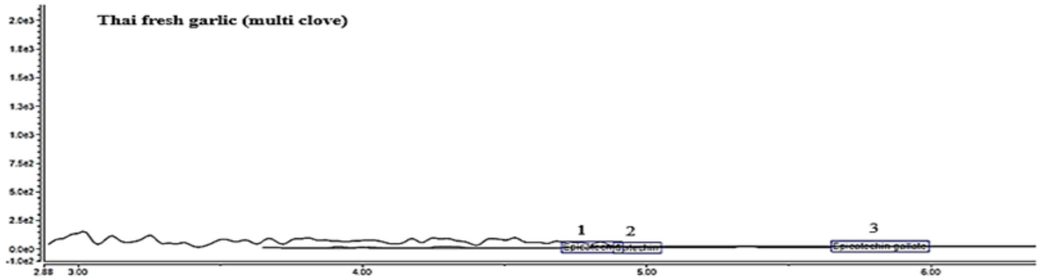

(**A**)

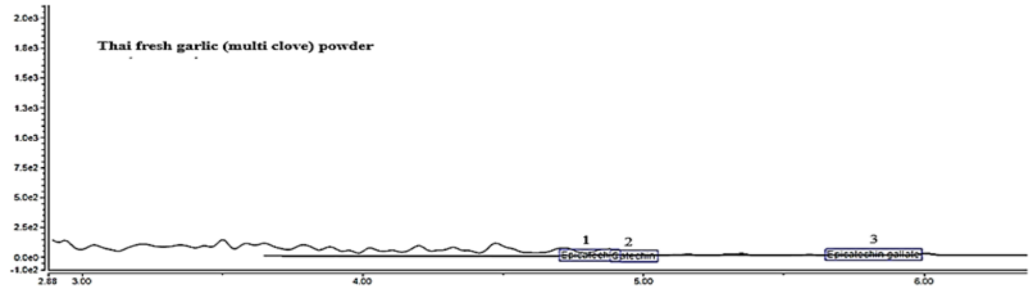

(**B**)

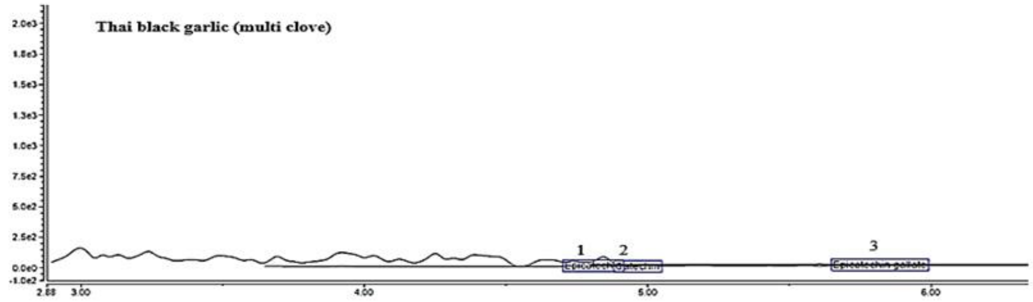

(**C**)

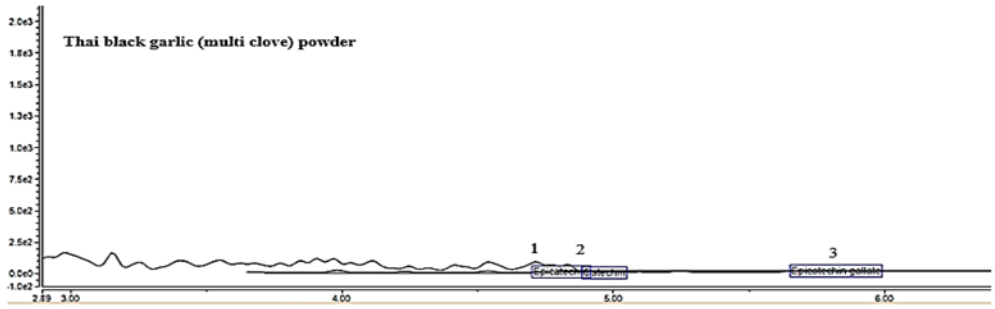

(**D**)

Figure S2. Detection of phenolic profiles of epicatechin (1), catechin (2) and epicatechin gallate (3) in Thai fresh garlic (multi clove) (A), Thai fresh garlic (multi clove) powder (B), Thai BG (C) and Thai BG powder (D) by LC-ESI-MS/MS.

Supplementary Figure S3:

The radical scavenging activity (% RSA) of Vitamin C (positive control) (A), Thai black garlic (B) and its powder (C)

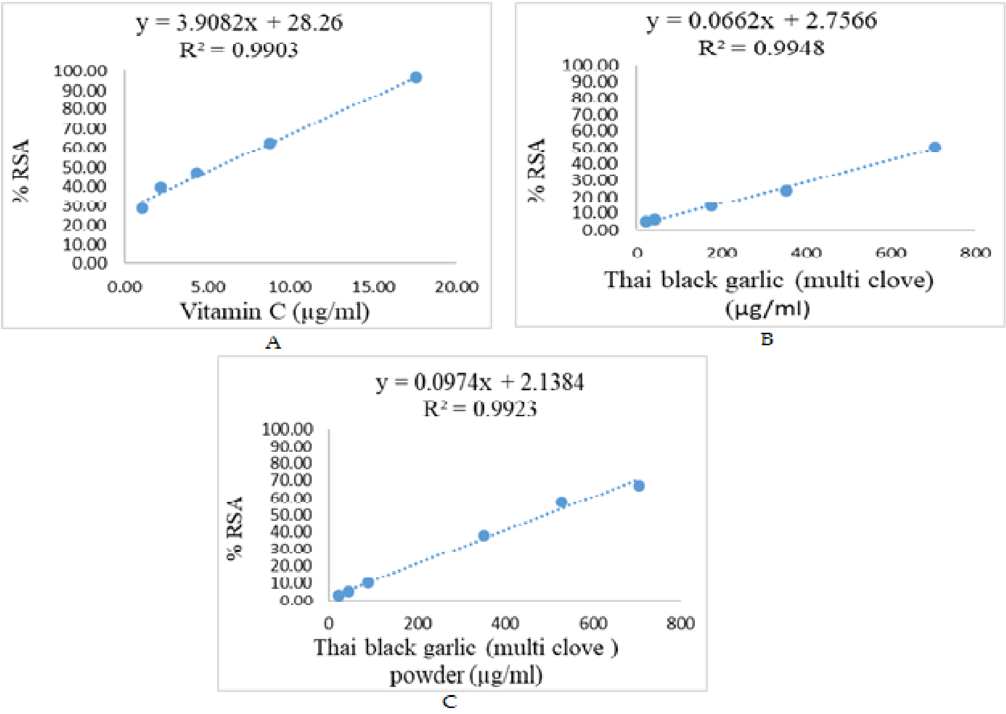

Figure S3. The radical scavenging activity (% RSA) of Vitamin C (positive control) (A), Thai BG (B) and Thai BG powder (C)
